# Effect of γ-aminobutyric Acid on Phenolics Metabolism in Barley Seedlings under Low NaCl Treatment

**DOI:** 10.3390/antiox10091421

**Published:** 2021-09-06

**Authors:** Mian Wang, Yahui Zhu, Pei Wang, Zhenxin Gu, Runqiang Yang

**Affiliations:** 1 College of Food Science and Technology, Whole Grain Food Engineering Research Center, Nanjing Agricultural University, Nanjing 210095, China; 2018108018@njau.edu.cn (M.W.); wangpei@njau.edu.cn (P.W.); guzx@njau.edu.cn (Z.G.); 2 College of Food Science and Technology, Tibet Agriculture and Animal Husbandry University, Linzhi 860000, China; zhuyahui@xza.edu.cn

**Keywords:** barley, GABA, phenolics, flavonoids, NaCl treatment

## Abstract

It has been revealed that high NaCl stress (>60 mmol L^−1^) induced phenolics accumulation in barley seedlings, with γ-aminobutyric acid (GABA) playing a key role. Interestingly, low NaCl stimulus (20 mmol L^−1^) enhancing phenolics synthesis and growth of barley seedlings was also reported recently. Hence, exogenous GABA and its bio-synthesis inhibitor 3-mercaptopropionic acid (3-MP) were applied to reveal the mechanism of GABA regulating phenolics metabolism in barley seedlings treated with 20 mmol L^−1^ NaCl. The contents of total phenolics and flavonoids significantly increased by 11.64% and 14.52% under NaCl, respectively. The addition of GABA further increased phenolics and flavonoids contents, especially for gallic acid, protocatechuic acid, caffeic acid, and quercetin, compared with NaCl treatment. Simultaneously, GABA increased the activities and mRNA levels of phenylalanine ammonia lyase (PAL), cinnamic acid 4-hydroxylase (C4H), and 4-coumalyl CoA ligase (4CL). The addition of 3-MP suppressed the above effects, except for increasing the protein levels of PAL, C4H, and 4CL. Low concentration of NaCl not only promoted growth, but also stimulated endogenous GABA metabolism to affect key enzymes activities and mRNA levels for phenolics synthesis in barley seedlings.

## 1. Introduction

Barley grains have the characteristics of high vitamins, high dietary fiber, and high protein contents, which could be stimulated by the accumulation of *γ*-aminobutyric acid (GABA), phenolics, and other nutrients by germination [1]. At present, a variety of barley seedling products have been launched globally, including Japanese “green juice”, compound powder tablets, solid drinks, cookies, and so on. GABA is a four-carbon non-protein amino acid found in prokaryotes and eukaryotes with the functions of lowering blood pressure, sedation, improving kidney and liver function, and promoting alcohol metabolism in the human body. It is mainly synthesized from glutamate catalyzed by glutamate dehydrogenase (GAD) in the cytoplasm of monocotyledonous plants [2], transported to mitochondria and degraded into succinic semialdehyde (SSA) by GABA aminotransferase (GABA-T). SSA is further oxidized to succinic acid by SSA dehydrogenase (SSADH) from the GABA shunt pathway [3]. GABA is necessary for carbon and nitrogen metabolism in plant growth and development under abiotic stress, including salt, hypoxia, and so on [4]. In addition, reports have shown that GABA also plays a role in signal transmission in plants under stress [5]. The GABA content in soybean increased by 25 times under salt and cold stress [6]. Mayer et al. [7] revealed that heat shock significantly enhanced the content of endogenous GABA in *Arabidopsis* gallnut cells.

Phenolics, including phenolic acids and flavonoids, are important secondary metabolites with anti-cancer, anti-tumor, anti-aging, and other health-promoting functions, which are mainly formed via the shikimate pathway and malonate pathway in plants [8]. The shikimic acid pathway is an important biological metabolic pathway connecting carbohydrate metabolism and aromatic compounds biosynthesis [9]. Phenylalanine ammonia lyase (PAL), cinnamic acid 4-hydroxylase (C4H), 4-coumalyl CoA ligase (4CL), chalcone isomerase (CHI), chalcone synthase (CHS), flavanone 3-hydroxylase (F3H), and anthocyanin synthase (ANS) are included in this pathway [10]. Among these, PAL is a key bridge connecting primary metabolism and phenylpropane metabolism [11]. Plants accumulate secondary metabolites such as phenolics in response to salt, low oxygen, low temperature, low pH, mechanical stimulation, heat shock, pathogenic microorganism infection, and so on [12]. The mechanism of phenolics accumulation in plants has been studied recently, mainly in relation to the activities and gene expressions of key enzymes involved in the phenolics synthesis pathway [13].

Exogenous GABA induced the synthesis of endogenous GABA and other free amino acids (especially proline) in barley seedlings with an increase of PAL, C4H, and 4CL activity, resulting in the enhancement of total phenolics and antioxidant capacity [14]. Adel et al. [15] found that phenolic substances in *Arabidopsis thaliana* were abundantly enriched under salt stress. Hela et al. [16] also found that total phenolics and flavonoids contents increased by 1.3 times and 14.5 times, respectively, in lettuce with 100 mmol L^−1^ NaCl treatment for 12 days. Both GABA and phenolics in barley seedlings could be accumulated under salt stress [14]. GABA has been confirmed to be a signal molecule involved in anabolic regulation under abiotic stress [17]. Ma et al. [14] found that GABA mediates phenolic compounds accumulation and antioxidant system enhancement in germinated hulless barley under NaCl stress (60 mM). However, higher NaCl concentration inhibited the growth and decreased the biomass of barley seedlings [13].

Interestingly, our recent study showed that low NaCl (20 mmol L^−1^) did not cause the stress effect on barley seedlings, but promoted growth compared with high salt stress, increasing total phenolics content and antioxidant capacity [18]. In addition, a large number of studies have concentrated on phenolic acids metabolism in cereals [19]. Flavonoids, as an important component of phenolics, are worthy of attention. Although the function of GABA in phenolic acids synthesis in barley seedlings under NaCl stress has been reported, the effects of GABA metabolism on phenolics accumulation under low NaCl treatment in barley seedlings is worthy of investigation, as it might be a different mode. Hence, the effects of GABA on the synthesis of phenolic acids and flavonoids in barley seedlings treated with a low concentration of NaCl were investigated on the physio-biochemistry and molecular levels.

## 2. Materials and Methods

### 2.1. Materials and Chemicals

Barley seeds were purchased from Jiangsu Yanjiang Institute of Agricultural Sciences, China, in July 2019. Ethyl acetate, linol, methanol, glutamic acid, and sodium hypochlorite were purchased from Sinopharm Chemical Reagent Company (Shanghai, China). Methanol (chromatographic grade), glacial acetic acid, acetonitrile, phenolic acids, and flavonoids were purchased from Sigma-Aldrich Chemical Co. (Shanghai, China). Trichloroacetic acid (TCA), 2-thiobarbituric acid (TBA), 3-mercaptopropionic acid (3-MP), 2,2′-diphenyl-1-picrylhydrazyl (DPPH), 2,2′-azino-bis (3-ethylbenzothiazoline-6-sulfonic acid) diammonium salt (ABTS), ATP, nicotinamide adenine dinucleotide phosphate (NADPH), ethylene diamine tetraacetic acid (EDTA), *β*-mercaptoethanol, coenzyme A (CoA), L-phenylalanine, trolox, GABA, dimethylaminobenzenesulfonyl chloride (derivatizing agent), and polyvinyl pyrrolidone (PVP) were purchased from Maclean Biochemical Technology Co., Ltd. (Shanghai, China). Hydrochloric acid and acetone were purchased from Nanjing Chemical Reagent Co., Ltd. (Nanjing, China).

### 2.2. Cultivation Condition and Treatments

The cultivation conditions and methods of the barley seedlings were based on the method used by Wang et al. [18]. Barley seeds were washed with ultrapure water and sterilized with 1% sodium hypochlorite solution for 15 min. After, the seeds were soaked at 25 °C for 6 h, and then cultivated at 25 °C in the dark.

CK (control): barley seedlings were growing in distilled water.

N: barley seedlings were growing in 20 mmol L^−1^ NaCl.

NG: barley seedlings were growing in 20 mmol L^−1^ NaCl containing 1.5 mmol L^−1^ GABA.

NM: barley seedlings were cultured with 35 μmol L^−1^ 3-MP under 20 mmol L^−1^ NaCl.

NMG: barley seedlings were cultured with 35 μmol L^−1^ 3-MP and 1.5 mmol L^−1^ GABA under 20 mmol L^−1^ NaCl.

Barley seedlings were sampled on day 6. One part of each sample was frozen in liquid nitrogen and stored at −80 °C; the other was vacuum freeze-dried and ground into powder using an electric mill (A11 Basic Analytical Mill, IKA, Guangzhou, Guangdong, China), then stored at −20 °C.

### 2.3. Determination of GABA Content, GAD, and GABA-T Activity

The GABA content was determined according to a reference method by Ma et al. [14]. From each freeze-dried sample, 0.5 g was taken. The samples, after being derivatized, were filtered through a 0.22 μm membrane filter for high-performance liquid chromatography (Agilent 1200, USA) analysis.

GAD activity was determined based on the method by Woodrow et al. [2] and appropriately adjusted. One gram of barley seedlings was ground homogeneously with 5 mL of 1/15 mol L^−1^ potassium phosphate buffer (pH 5.8), containing 2 mol L^−1^ *β*-mercaptoethanol, 2 mmol L^−1^ EDTA, and 0.2 mmol L^−1^ PLP. After being centrifuged (10,000× *g*, 4 °C for 20 min), the supernatant (200 μL) was mixed with 100 μL of l% Glu (pH 5.8) at 40 °C for 2 h, then terminated at 90 °C. The samples were precipitated overnight with 5 mL of absolute ethanol at 4 °C, then the above method for GABA was followed. The product GABA content (1 μmol GABA per 1 h) was determined as one enzyme unit.

GABA-T activity was determined by Plant GABA-T ELISA kit (Jianchen, Nanjing, China), and specific steps were followed as per the instructions.

### 2.4. Determination of Basic Physiological Index

The length of seedlings was determined by selecting 30 barley seedlings and directly measuring the length of the terminal or radical bud with a vernier caliper. Ratio of fresh to dry weight in barley seedlings was determined by measuring the weight of 50 seedlings before and after vacuum freeze-drying. Respiratory rate was measured using a slightly modified version of the method described by He et al. [20]. Malondialdehyde (MDA) content determination was based on a reference method by Alquraan and Alomari [21] with appropriate adjustments. Briefly, 1.0 g of barley seedlings was ground with 5 mL of 10% TCA. After being centrifuged (10,000× *g*, 20 min), the supernatant was boiled with 0.67% TBA (1:1 *v*/*v*) for 30 min, pre-cooled, and centrifuged (4000× *g*, 15 min). The supernatant was measured at 450 nm, 532 nm, and 600 nm using a spectrophotometer (Uniko instrument co., Ltd., Shanghai, China). For electrolyte permeability determination, approximately 1.0 g of barley seedlings was cut into 3 mm length, and shaken with 30 mL distilled water at room temperature for 1 h; then, the conductivity was measured as EC_1_. The reaction solution was bathed at 90 °C for 15 min to kill plant tissue. After returning it to room temperature, the reaction solution was topped up to 30 mL with distilled water, the conductivity of which was measured as EC_2_. $Electrolyte\;permeability\left(\%\right)=\frac{EC_{1}}{EC_{2}}\times 100\%$.

### 2.5. Determination of Total Phenolics and Flavonoids Contents

Free and bound phenolic contents were measured according to the method described by Wang et al. [18]. A freeze-dried sample (1.0 g) was extracted with 80% methanol (1:20 *m*/*v*) and stored with 50% methanol at −20 °C as free phenolic solution (FPS). After the extraction of FPS, the residue was hydrolyzed with 2 mol L^−1^ NaOH at 25 °C for 4 h, then adjusted to pH 1.5–2.0 with HCl. The sample was extracted with ethyl acetate and stored with 50% methanol at −20 °C as bound phenolic solution (BPS).

Total phenolics content was determined according to the method described by Islam et al. [19]. The standard was gallic acid. Results were expressed as milligrams of gallic acid equivalents per 100 g of dry weight (DW). Total flavonoids content was determined according to the method described by Islam et al. [22]. The standard was rutin. Results were expressed as milligrams of rutin equivalents per 100 g of DW.

### 2.6. Quantification of Phenolic Acids and Flavonoids

FPS and BPS were filtered through a 0.45 μm membrane filter for high-performance liquid chromatography (LC-20A; Shimadzu, Kyoto, Japan) analysis. Phenolic and flavonoid compounds of 6-day-old barley seedlings were measured according to the method described by Chen et al. [23], with appropriate adjustments. A reversed phase column (Shimadzu C18 110A, 4.6 × 150 mm, 5 μm particle size) was used. Mobile phase A consisted of 0.1% acetic acid in water, and mobile phase B consisted of 0.1% acetic acid in methanol. The HPLC conditions were identical to those of Wang et al. [18].

### 2.7. Determination of Key Enzymes Activities in Phenolics Synthesis

The measurement of PAL activity referred to the method of Khademi et al. [24]. Determination of C4H activity referred to the method of Ma et al. [13]. Determination of 4CL activity was based on the method of Yan et al. [25]. Barley seedlings were pre-frozen with liquid nitrogen and stored at −80 °C. The measurement methods were adjusted appropriately according to the experimental needs.

### 2.8. Determination of ABTS and DPPH Radical Scavenging Activities

This was measured according to the method described by Islam et al. [3], with trolox as the standard.

### 2.9. mRNA Levels Analysis

Total RNA isolation and real-time PCR (polymerase chain reaction) were conducted according to Wang et al. [18]. The primers sequences of barley *PAL*, *C4H,* and *4CL* (Table 1) for real-time PCR analysis were designed using Primer 5.0 primer design software according to the barley-related target gene nucleic acid sequence published on NCBI, and synthesized by GenScript Biotechnology Co., Ltd. (Nanjing, China). Total RNA was extracted from barley seedlings with Plant RNA Extraction Kit (Takara, cat. #9769). The reverse transcription and fluorescence quantitative analysis were performed using RT-PCR Mater Mix Kit (Takara, cat. #RR036A) and SYBR Premix Ex Taq^TM^ Kit (Takara, cat. #RR420A) according to the instructions.

### 2.10. Western Blot

The 6-day-old barley seedlings used for Western blot assays and the specific operation process were based on the method of Wang et al. [18]. Total plant protein was extracted using Tissue Protein Extraction Reagent (CAT# 78510; Thermal Scientific, Shanghai, China). The content of protein was quantified using a BCA protein assay kit (CAT# P0012S; Beyotime Biotechnology, Shanghai, China). Proteins were denatured with 1×SDS loading buffer at 90 °C for 10 min. Then, proteins were electrophoresed on polyacrylamide gels and electro-transferred to mini-size PVDF membranes (cat. #IPVH00010; Millipore, Shanghai, China). Antibodies of PAL, C4H, 4CL, and rubisco (Nanjing Aoqing Bio-Tech. Co. Ltd., Nanjing, China) were diluted according to the instructions. Immune complexes detection was performed with a horseradish peroxidase-conjugated secondary antibody (1:5000, cat. #31431; Thermal Scientific, Shanghai, China).

### 2.11. Statistical Analysis

Experimental data were expressed as mean ± standard deviation (SD) with three or more replications (*n* ≥ 3). SPSS 18.0 (SPSS Inc., Chicago, IL, USA) was applied for significant difference tests. Data were analyzed by Duncan’s multiple-range tests at *p* < 0.05.

## 3. Results

### 3.1. GABA Metabolism of Barley Seedlings

**Figure 1:**
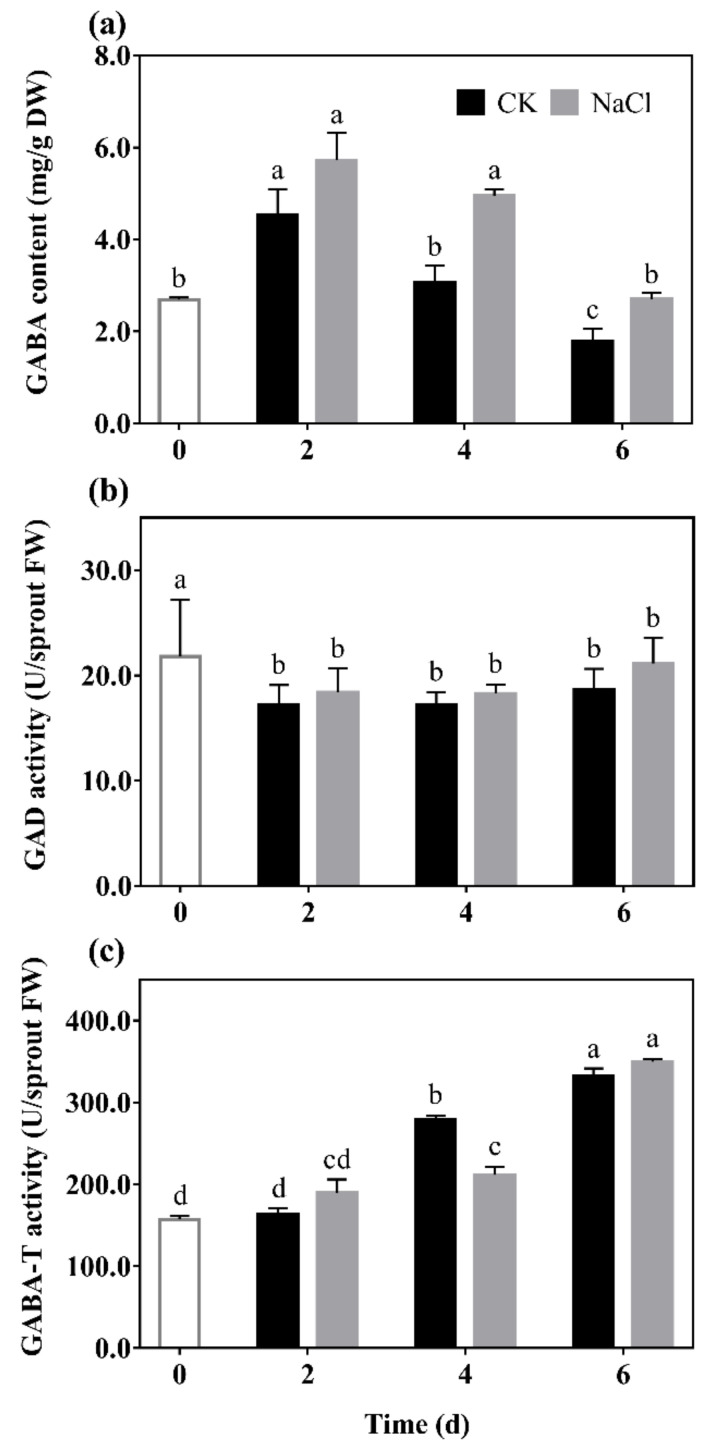
The effect of NaCl on GABA content (**a**), GAD activity (**b**), and GABA-T activity (**c**) of barley seedlings. Barley seedings were cultured under CK and N treatments, respectively. Sampling was performed on day 0, 2, 4, and 6, respectively. Bars represent standard deviation of means (*n* = 3), and means with different lower case letters were significantly different (*p* < 0.05). Two-way ANOVA was used.

GABA content (a), GAD (b), and GABA-T (c) activity of barley seedlings treated with 20 mM NaCl are shown in Figure 1. It can be seen that the GABA content of barley seedlings increased to its maximum on the 2nd day, which was 1.25 times that of the control. GABA content gradually decreased with the increase of germination days under N treatment, which always higher than the control (Figure 1a).

The results show that there was a downward trend in the GAD activity of barley seedlings with the increase of germination days (Figure 1b). Among them, GAD activity under N treatment was significantly increased by 14.84% and 36.59% compared with the control on the 2nd and 6th days, respectively. The GABA-T activity of the barley seedlings significantly decreased by 37.82% compared with the control on the 4th day, and there was no significant difference on the other days.

Results indicated that low salt stimulation could significantly promote endogenous GABA synthesis and regulate GABA metabolism in barley seedlings.

### 3.2. Physiological Indicators of Barley Seedlings

**Figure 2:**
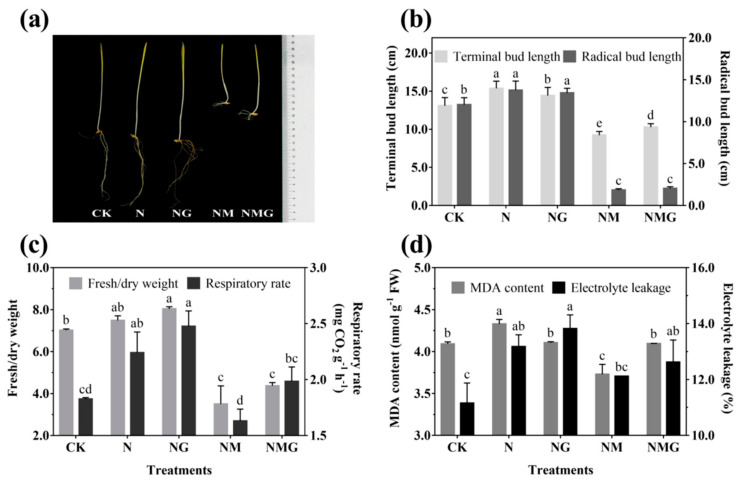
The effect of GABA on the growing status (**a**), terminal/radical bud length (**b**), ratio of fresh to dry weight and respiratory rate (**c**), and MDA content and electrolyte leakage (**d**) of 6-day-old barley seedlings under 20 mmol L^−1^ NaCl. The lower case letters for each index indicate significant differences at *p* < 0.05 among different treatments. One-way ANOVA was used for data analysis. The data were presented as mean ± SD, *n* ≥ 4.

The morphology of 6-day-old barley seedlings under different treatments is shown in Figure 2a. The terminal and radical bud length increased significantly under NaCl (N) and NaCl + GABA (NG) treatments (Figure 2b). The growth of barley seedlings was inhibited by 3-MP treatment (NM), especially the radical length, but the terminal length was improved by the addition of GABA (NMG) (Figure 2a,b). NaCl and GABA not only promoted the growth of barley seedlings, but also increased their ratio of fresh to dry weight and their respiration rate (Figure 2c). The ratio of fresh to dry weight and the respiration rate of barley seedlings treated with GABA were much higher than the others, which were significantly increased by 13.65% and 35.52% compared with the control (CK), respectively. Oxidative damage of barley seedlings was deepened by NaCl (Figure 2d), mainly manifested by the increase of MDA content and electrolyte leakage. However, there was no significant difference in the MDA content of barley seedlings under NG compared with CK, while electrolyte leakage was significantly increased by 23.84%. In short, NaCl and exogenous GABA could stimulate the growth of barley seedlings, increase the permeability of cell membranes, and accelerate cell growth and metabolism.

### 3.3. The Contents and Antioxidant Capacities of Total Phenolics and Flavonoids in Barley Seedlings

**Figure 3:**
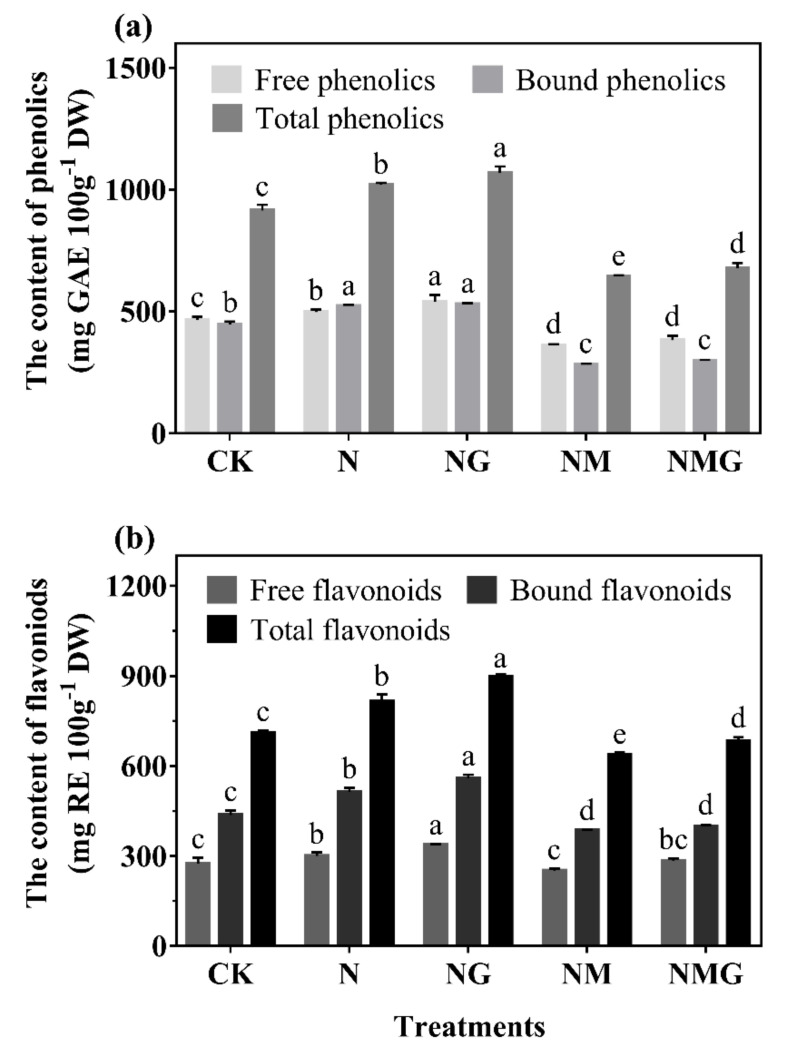
The effect of GABA on phenolics (**a**) and flavonoids (**b**) contents in 6-day-old barley seedlings under 20 mmol L^−1^ NaCl. The lower case letters indicate significant differences at *p* < 0.05 among different treatments. One-way ANOVA was used. The data are presented as mean ± SD, *n* ≥ 3.

The contents of total phenolics in barley seedlings under N and NG treatments increased by 11.64% and 17.00%, respectively, compared with CK (Figure 3a). Free and bound phenolics contents increased by 6.14% and 16.74%, respectively, under N treatment compared with CK. Added GABA further increased free and bound phenolics contents by 15.67% and 18.38% (NG). Phenolics content under NM treatment was significantly lower than that under NaCl, but the further addition of GABA increased phenolics content very little (NMG). As shown in Figure 3b, flavonoids mostly presented in the bound state in barley seedlings, which significantly increased under both NaCl and GABA. Free, bound, and total flavonoids contents of barley seedlings significantly increased by 9.56%, 17.63%, and 14.52% under 20 mmol L^−1^ NaCl, respectively, compared with CK. The further added GABA increased their contents by 22.80%, 28.11%, and 26.06%, respectively. The effects of NM and NMG treatments on the content of flavonoids showed a similar trend to that of phenolics.

**Figure 4:**
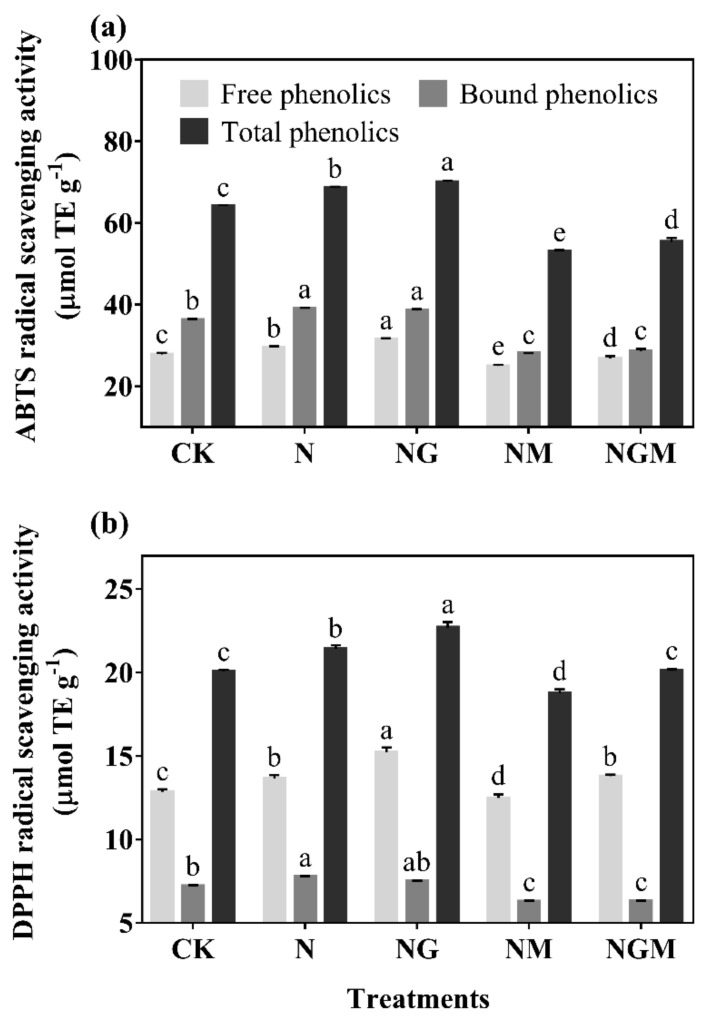
The effect of GABA on the ABTS (**a**) and DPPH (**b**) radical scavenging capacities in 6-day-old barley seedlings under 20 mmol L^−1^ NaCl. The lower case letters indicate significant differences at *p* < 0.05 among different treatments. One-way ANOVA was used. The data are presented as mean ± SD, *n* = 3.

The antioxidant capacities of free, bound, and total phenolics of 6-day-old barley seedlings under N and NG treatment significantly improved with a large amount of phenolics (Figure 4). ABTS free radical scavenging capacity of total phenolics increased by 6.90% and 9.28% under N and NG treatments compared with the control, respectively, while DPPH free radical scavenging ability increased by 6.78% and 13.16% compared with the control, respectively. In addition, bound phenolics had a greater contribution to ABTS free radical scavenging ability, while free phenolics had a greater contribution to DPPH free radical scavenging ability.

### 3.4. The Composition and Contents of Phenolics Acids and Flavonoids in Barley Seedlings

Nine major phenolic acids were detected (gallic acid, protocatechuic acid, *p*-hydroxybenzoic acid, vanillic acid, caffeic acid, syringic acid, *p*-coumaric acid, ferulic acid, and sinapinic acid). As shown in Table 2, the composition and content of individual phenolic acids changed to different degrees under each treatment. Among them, gallic acid, protocatechuic acid, and syringic acid mainly existed in free form, while *p*-coumaric acid and ferulic acid basically existed in bound form. Gallic acid, protocatechuic acid, *p*-hydroxybenzoic acid, caffeic acid, syringic acid, and sinapinic acid showed relatively higher contents in free state under N and NG treatments compared with CK, while free vanillic acid content decreased significantly. The contents of bound vanillic acid and bound ferulic acid increased significantly under N and NG treatments compared with CK. In addition, the content of bound caffeic acid significantly increased under NG treatment. Free *p*-coumaric acid and ferulic acid were detectable under NG treatment, while vanillic acid and *p*-coumaric acid showed relatively lower contents compare with CK. The contents of gallic acid, *p*-hydroxybenzoic acid, vanillic acid, *p*-coumaric acid, ferulic acid, and sinapinic acid decreased significantly under NM treatment, especially free ferulic acid, free *p*-hydroxybenzoic acid, and bound *p*-coumaric acid. The further added GABA restored the contents of free gallic acid, free protocatechuic acid, bound ferulic acid, and bound caffeic acid to a certain extent. In summary, NaCl and GABA enhanced the synthesis of phenolic acid of barley seedlings to varying degrees. In addition, GABA could effectively alleviate the inhibitory effect of 3-MP on phenolic acid synthesis.

Five major flavonoids were detected (catechinic acid, fisetin, myricetin, quercetin, and apigenin). Both N and NG treatments significantly stimulated flavonoids synthesis in seedlings, but the composition and content of individual flavonoids were different. Fischerin, myricetin, and quercetin were mainly presented as bound form. Fischerin and myricetin contents increased significantly under N and NG treatments. Catechinic acid, fisetin, myricetin, and apigenin increased by 52.75%, 76.60%, 25.61%, 9.16%, and 58.25% under NG treatment, respectively, compared with CK. The content of free catechinic acid increased significantly, while the content of bound catechinic acid decreased under NG treatment. The contents of catechins, fisetin, myricetin, and quercetin decreased significantly under NM treatment, while bound catechins and myricetin were not detectable. The added GABA further significantly increased bound myricetin content.

### 3.5. The Enzyme Activity and Gene Expression Related to Phenolics Synthesis in Barley Seedlings

**Figure 5:**
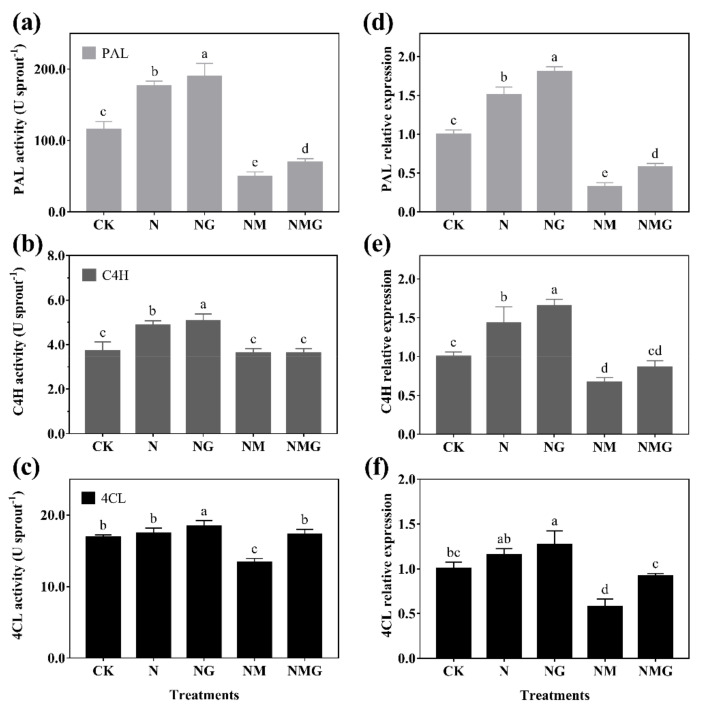
The effect of GABA on PAL activity (**a**), C4H activity (**b**), and 4CL activity (**c**), and their gene expression (**d**–**f**), for 6-day-old barley seedlings under 20 mmol L^−1^ NaCl. The lower case letters indicate significant differences at *p* < 0.05 among different treatments. One-way ANOVA was used. The data are presented as mean ± SD, *n* = 4.

The activity of PAL, C4H, and 4CL were enhanced to different degrees under N and NG treatments. Among them, PAL activity significantly increased by 52.40% and 63.93% compared with CK, respectively (Figure 5a). C4H activity increased by 31.00% and 36.66%, respectively (Figure 5b). 4CL activity did not change significantly under N treatment, but it increased by 8.86% under NG treatment compared with CK (Figure 5c). The trends in expressions of *PAL*, *C4H,* and *4CL* were basically consistent with their activities (Figure 5d–f). In addition, the activities and gene expression of PAL and 4CL decreased significantly under NM treatment, and improved to varying degrees under NMG treatment. Results show that the expressions of *PAL*, *C4H,* and *4CL* in barley seedlings were mainly regulated by NaCl and GABA, stimulating the enzyme activities to accumulate phenolics.

### 3.6. The Protein Expression of PAL, C4H, and 4CL in Barley Seedlings

**Figure 6:**
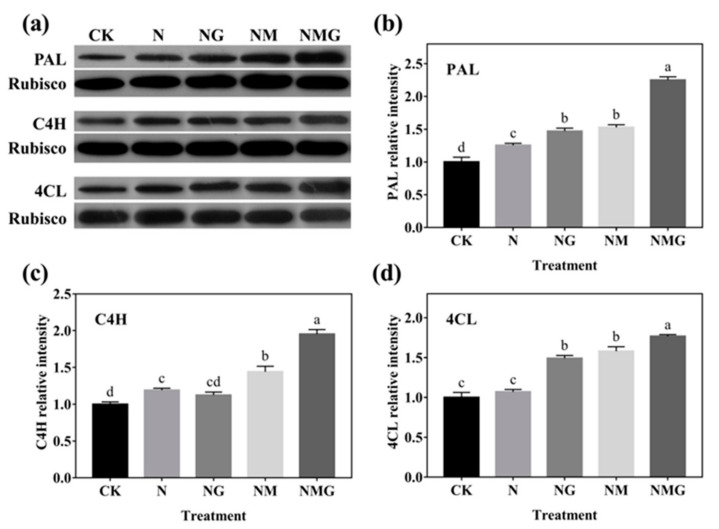
The effect of GABA on PAL, C4H, and 4CL protein expression of 6-day-old barley seedlings under 20 mmol L^−1^ NaCl. Panels show representative bands (**a**). Histograms represent relative protein levels of PAL (**b**), C4H (**c**) and 4CL (**d**) of 6-day-old barley seedlings normalized to the corresponding rubisco in different treatments. The lower case letters indicate significant differences at *p* < 0.05 among different treatments. One-way ANOVA was used. The data are presented as mean ± SD, *n* = 3.

The relative protein expression of key enzymes for phenolics synthesis is shown in Figure 6. The relative expression levels of PAL and C4H in barley seedlings under N treatment significantly increased by 25% and 19.33% compared with CK. The PAL, C4H, and 4CL relative expression levels under NG treatment increased, significantly, by 47.13%, 12.44%, and 49.02% compared with CK as well. Unexpectedly, the protein expression of three enzymes was further increased under the NM and NMG treatments, which was inconsistent with the trends of their activities and gene expression.

## 4. Discussion

There have been a large number of studies on the metabolism and accumulation of GABA under salt stress. It has been found that high concentrations of salt increased GABA content, but inhibited the growth of plants at the same time [17]. However, this study showed that low NaCl concentration (20 mmol L^−1^) did not inhibit the growth of barley seedlings, but promoted the growth status (Figure 2). Simultaneously, the endogenous GABA level significantly increased (Figure 1a), due to a higher level of GAD activity compared with CK (Figure 1b). GABA, as a signal molecule, has been widely studied in plant stress signal transmission [2]. This study found that total phenolics and flavonoids contents were significantly increased by GABA under N treatment, and decreased by 3-MP. The further added GABA had a certain recovery effect on total phenolics and flavonoids contents (Figure 3), indicating that GABA participated in the synthesis of phenolics under N treatment.

Phenolics are mainly distributed in the cortex of plants, combined with cellulose, hemicellulose, and other non-starch polysaccharides [12], which play a critical role in resisting adversity and protecting plants from abiotic and biotic stress [13]. The synthesis of phenolics is closely related to the growth of plants. NaCl (20 mmol L^−1^) significantly promoted the growth of barley seedlings and increased phenolics content. GABA further promoted the above effects under N treatment (Figure 2 and Figure 3), indicating that GABA promoting the accumulation of phenolics was primarily related to the promotion of growth of barley seedlings. Total phenolics and flavonoids contents of barley seedlings increased significantly by 11.64% and 14.52% under N treatment, respectively (Figure 3), and led to an increase of ABTS and DPPH free radical scavenging ability (Figure 4). As a signal molecule, GABA plays a key role in the response of plants to external stimuli. Under salt stress, GABA accumulated in germinated soybean [4], faba bean [26], and brown rice [27]. At the same time, GABA promoted the synthesis of phenolics under NaCl stress (60 mmol L^−1^) in barley seedlings [13]. Although low concentrations of NaCl promoted the growth and biomass accumulation of barley seedlings in the present study, MDA content and electrical leakage also increased significantly (Figure 2d), indicating that low concentration of NaCl also slightly caused damage to the tissue and cells of seedlings. Simultaneously, the synthesis of endogenous GABA was also activated (Figure 1), and GABA participated in phenolics synthesis of barley seedlings stimulated by low concentration of NaCl (20 mmol L^−1^).

As the main phenolics in barley seedlings, the composition and contents of phenolic acids and flavonoids were affected by GABA. GABA mainly increased the contents of bound phenolic acids and flavonoids under N treatment, especially for caffeic acid, ferulic acid (Table 2), fisetin, myricetin, and quercetin (Table 3). It showed that GABA had different effects on the existence and contents of various phenolic acids and flavonoids. However, 3-MP significantly decreased the contents of bound phenolic acids and flavonoids, especially bound protocatechuic acid, *p*-hydroxybenzoic acid, and catechinic acid, illustrating that endogenous GABA had an important effect on the synthesis of phenolics in barley seedlings treated with NaCl, which improved the resistance to abiotic stress of plants [2]. In this study, there was no stress under 20 mmol L^−1^ NaCl, so the mechanism of GABA promoting phenolics and enhancing antioxidant capacity was different. GABA promoted the growth of barley seedlings, increasing the metabolism rate of cells and demand for material energy, resulting in the increased demand for plant cell wall components, including phenolic acids and flavonoids, leading to the synthesis of large amounts of phenolics.

In addition, PAL, C4H, and 4CL are three key rate-limiting enzymes of the phenylpropane metabolism pathway. Their activity and expression determine the synthesis of phenolics. In this study, PAL and C4H activity were enhanced significantly by GABA in barley seedlings under N treatment, which was basically consistent with their gene and protein expression (Figure 5 and Figure 6). Interestingly, 3-MP suppressed the activities of PAL, C4H, and 4CL, while increasing their protein expression, because 3-MP caused damage to cells, which broke the balance of osmotic pressure and inhibited the growth of seedlings. Simultaneously, the activities and mRNA levels of PAL, C4H, and 4CL decreased significantly. More antioxidants were needed because the biosynthesis of phenolics synthesis was inhibited by 3-MP, which might be induced by NaCl.

## 5. Conclusions

GABA could not only enhance the activities and expressions of PAL, C4H, and 4CL involved in the phenylpropane metabolism pathway to accumulate phenolics, but also stimulated the growth and metabolism of barley seedlings, and could be synthesized in large quantities in barley seedlings treated with low NaCl (20 mmol L^−1^).

## Figures and Tables

**Table 1:** Primers used in this study.

Gene	Primer Name	Primer Sequences (5′→3′)	Access No.
PAL	Sense	CACTGAATGCCGATCATACCC	AK250100.1
	Ant-sense	CCGTTCCAACCCTTGAGACA	
C4H	Sense	CGTACGTGCTCTCGGAGTTC	KF927086.1
	Ant-sense	GTCTTTCCTCCCCGTTGGAC	
4CL	Sense	GGTGGAGATCGCCAAGAGCC	KF442977.1
	Ant-sense	CTCCGTCATCCCGTACCCCT	
Actin	Sense	TCGTGAGAAGATGACCCAGA	AK251023.1
	Ant-sense	CCGAGTCCAGCACAATACCT	

**Table 2:** The individual phenolic acid content in barley seedlings.

Type	Treatments	Content (µg g^−1^ DW)		
		Free	Bound	Total
Gallic acid	CK	12.40 ± 0.20 ^e^	ND	12.40 ± 0.20 ^e^
	N	258.52 ± 2.95 ^c^	ND	258.52 ± 2.95 ^c^
	NG	361.63 ± 1.04 ^a^	ND	361.63 ± 1.04 ^a^
	NM	212.65 ± 3.38 ^d^	ND	212.65 ± 3.38 ^d^
	NMG	281.46 ± 0.65 ^b^	ND	281.46 ± 0.65 ^b^
Protocatechuic acid	CK	320.70 ± 22.57 ^c^	5.81 ± 0.21 ^a^	326.50 ± 22.79 ^c^
	N	ND	5.37 ± 0.09 ^b^	5.37 ± 0.09 ^d^
	NG	622.01 ± 12.53 ^b^	5.90 ± 0.06 ^a^	627.90 ± 12.58 ^b^
	NM	597.17 ± 40.89 ^b^	ND	597.17 ± 40.89 ^b^
	NMG	692.15 ± 5.09 ^a^	ND	692.15 ± 5.09 ^a^
*p*-Hydroxybenzoic acid	CK	6.10 ± 0.15 ^b^	3.56 ± 0.05 ^c^	9.66 ± 0.20 ^c^
	N	11.08 ± 0.48 ^a^	4.56 ± 0.01 ^b^	15.64 ± 0.47 ^b^
	NG	11.42 ± 0.17 ^a^	5.45 ± 0.04 ^a^	16.87 ± 0.21 ^a^
	NM	5.39 ± 0.03 ^c^	ND	5.39 ± 0.03 ^d^
	NMG	5.83 ± 0.05 ^bc^	ND	5.83 ± 0.05 ^d^
Vanillic acid	CK	23.27 ± 0.12 ^a^	11.33 ± 0.10 ^c^	34.61 ± 0.02 ^a^
	N	9.64 ± 0.62 ^b^	13.04 ± 0.13 ^b^	22.68 ± 0.75 ^b^
	NG	5.26 ± 0.50 ^c^	15.20 ± 0.12 ^a^	20.46 ± 0.37 ^c^
	NM	ND	6.51 ± 0.03 ^d^	6.51 ± 0.40 ^e^
	NMG	3.92 ± 0.19 ^d^	5.87 ± 0.40 ^e^	9.79 ± 0.22 ^d^
Caffeic acid	CK	9.75 ± 0.01 ^c^	13.16 ± 0.06 ^b^	22.90 ± 0.07 ^c^
	N	13.75 ± 0.02 ^b^	13.18 ± 0.25 ^b^	26.92 ± 0.27 ^b^
	NG	15.20 ± 0.07 ^a^	104.20 ± 1.42 ^a^	119.40 ± 1.49 ^a^
	NM	9.04 ± 0.02 ^d^	7.32 ^d^	16.36 ± 0.02 ^e^
	NMG	8.54 ± 0.08 ^e^	10.13 ± 0.08 ^c^	18.67 ^d^
Syringic acid	CK	1.08 ± 0.01 ^c^	ND	1.08 ± 0.01 ^c^
	N	2.51 ± 0.02 ^a^	ND	2.51 ± 0.02 ^a^
	NG	2.34 ± 0.04 ^b^	ND	2.34 ± 0.04 ^b^
	NM	ND	ND	ND
	NMG	ND	ND	ND
*p*-Coumaric acid	CK	ND	508.30 ± 0.97 ^b^	508.30 ± 0.97 ^b^
	N	ND	521.87 ± 0.42 ^a^	521.87 ± 0.42 ^a^
	NG	2.47 ± 0.08 ^a^	470.17 ± 1.75 ^c^	472.64 ± 1.83 ^c^
	NM	ND	69.02 ± 0.82d ^e^	69.02 ± 0.82 ^e^
	NMG	ND	74.88 ± 1.00 ^d^	74.88 ± 1.00 ^d^
Ferulic acid	CK	ND	2463.52 ± 2.59 ^b^	2463.52 ± 2.59 ^b^
	N	ND	3129.04 ± 6.19 ^a^	3129.04 ± 6.19 ^a^
	NG	2.48 ± 0.01 ^a^	3144.07 ± 9.93 ^a^	3146.55 ± 9.93 ^a^
	NM	ND	1448.45 ± 14.51 ^d^	1448.45 ± 14.51 ^d^
	NMG	ND	1632.20 ± 13.02 ^c^	1632.20 ± 13.02 ^c^
Sinapinic acid	CK	30.17 ± 0.02 ^c^	30.11 ± 0.17 ^b^	60.28 ± 0.19 ^c^
	N	39.03 ± 0.42 ^a^	34.47 ± 0.60 ^a^	73.50 ± 1.02 ^a^
	NG	37.20 ± 0.39 ^b^	34.39 ± 0.17 ^a^	71.58 ± 0.21 ^b^
	NM	ND	30.25 ± 0.09 ^b^	30.25 ± 0.09 ^d^
	NMG	ND	28.53 ± 0.68 ^c^	28.53 ± 0.68 ^e^

The individual phenolic acid content of 6-day-old barley seedlings is presented here. The lower case letters in the same column for each phenolic acid indicate significant differences at *p* < 0.05 among different treatments. One-way ANOVA was used. ND means “not detectable”. The data are presented as mean ± SD, *n* ≥ 3.

**Table 3:** The individual flavonoid content in barley seedlings.

Type	Treatments	Content (µg g^−1^ DW)		
		Free	Bound	Total
Catechinic acid	CK	22.90 ± 0.87 ^b^	23.43 ^b^	46.33 ± 0.86 ^b^
	N	24.17 ± 0.36 ^b^	24.49 ± 0.13 ^a^	48.66 ± 0.23 ^b^
	NG	49.06 ± 1.96 ^a^	21.71 ± 0.32 ^c^	70.77 ± 2.29 ^a^
	NM	19.98 ± 0.24 ^c^	ND	19.98 ± 0.24 ^c^
	NMG	19.85 ± 0.37 ^c^	ND	19.85 ± 0.37 ^c^
Fisetin	CK	159.78 ± 0.33 ^d^	326.62 ± 10.99 ^c^	486.40 ± 10.67 ^c^
	N	166.63 ± 1.42 ^c^	593.28 ± 10.52 ^b^	759.92 ± 11.94 ^b^
	NG	201.63 ± 2.61 ^a^	657.37 ± 6.68 ^a^	859.00 ± 9.29 ^a^
	NM	179.71 ± 2.21 ^b^	196.24 ± 0.83 ^d^	375.95 ± 1.38 ^d^
	NMG	156.69 ± 0.39 ^d^	203.00 ± 3.37 ^d^	359.69 ± 3.76 ^d^
Myricetin	CK	44.68 ± 0.69 ^b^	321.30 ± 0.80 ^b^	365.99 ± 1.50 ^c^
	N	44.60 ± 0.83 ^b^	415.11 ± 12.03 ^a^	459.71 ± 12.86 ^b^
	NG	53.26 ± 3.47 ^a^	425.13 ± 3.39 ^a^	478.39 ± 6.85 ^a^
	NM	33.83 ± 0.36 ^c^	ND	33.83 ± 0.36 ^e^
	NMG	ND	154.85 ± 6.71 ^c^	154.85 ± 6.71 ^d^
Quercetin	CK	ND	496.19 ± 11.89 ^b^	496.19 ± 11.89 ^b^
	N	ND	452.24 ± 10.48 ^c^	452.24 ± 10.48 ^c^
	NG	ND	541.64 ± 9.71 ^a^	541.64 ± 9.71 ^a^
	NM	ND	164.75 ± 5.84 ^d^	164.75 ± 5.84 ^d^
	NMG	ND	158.24 ± 4.78 ^d^	158.24 ± 4.78 ^d^
Apigenin	CK	0.057 ± 0.001 ^d^	0.707 ± 0.049 ^c^	0.764 ± 0.048 ^c^
	N	0.096 ± 0.002 ^b^	0.997 ± 0.025 ^b^	1.093 ± 0.027 ^b^
	NG	0.110 ± 0.006 ^a^	1.099 ± 0.005 ^a^	1.209 ± 0.001 ^a^
	NM	0.069 ± 0.002 ^c^	0.270 ± 0.009 ^e^	0.340 ± 0.007 ^d^
	NMG	ND	0.368 ± 0.002 ^d^	0.368 ± 0.002 ^d^

The individual flavonoid content of 6-day-old barley seedlings. The lower case letters in the same column for each flavonoid indicate significant differences at *p* < 0.05 among different treatments. One-way ANOVA was used. ND means “not detectable”. The data are presented as mean ± SD, *n* ≥ 3.

## Data Availability

The data presented in this study are available in this manuscript.
